# Lactylation in Cancer: Unlocking the Key to Drug Resistance and Therapeutic Breakthroughs

**DOI:** 10.32604/or.2025.067343

**Published:** 2025-10-22

**Authors:** Xiangnan Feng, Dayong Li, Pingyu Wang, Xinyu Li, Guangyao Li

**Affiliations:** 1 Clinical School of Medicine, Anhui Medical University, Hefei, 230022, China; 2 Department of Gastrointestinal Surgery, The Second People’s Hospital of Wuhu, Wuhu, 241000, China

**Keywords:** Lactylation, drug resistance, cancer metabolism, tumor microenvironment, immune evasion

## Abstract

Lactylation, a post-translational modification process that adds lactate groups to lysine residues, plays a crucial role in cancer biology, especially in drug resistance. However, the specific molecular mechanisms of lactylation in cancer progression and drug resistance are still unclear, and therapeutic strategies targeting the lactylation pathway are expected to overcome metabolic reprogramming and immune evasion. Therefore, this article provides a comprehensive description and summary of lactylation modification and tumor drug resistance. Numerous studies have shown that, due to the Warburg effect, there is an abnormally high level of lactate in tumor cells. Elevated levels of lactate promote metabolic reprogramming and alter key cellular processes, including gene expression, DNA repair, and immune regulation. These cellular processes are precisely the key factors for tumor cells to develop drug resistance. Lactylation also affects the tumor microenvironment, promoting immune evasion and resistance to immunotherapy in tumor cells. This modification affects proteins involved in metabolic pathways, glycolysis, and mitochondrial function, further supporting tumor growth and metastasis. Therefore, this article provides a comprehensive description and summary of lactylation modification and tumor drug resistance to clarify the specific mechanisms between the two and provide references and directions for future research on tumor drug resistance.

## 1 Introduction

Cancer is a major global health concern, with increasing morbidity and mortality rates worldwide [1]. As an age-related disease, cancer poses a particular threat to China, which has the world’s largest elderly population (≥60 years) and is experiencing an unprecedented population ageing [2]. As a result, cancer has become one of the most common causes of death in China [2,3]. At present, the main treatments for cancer include surgery, radiotherapy, and chemotherapy, such as hepatocellular carcinoma (HCC), which is the third leading cause of cancer-related deaths worldwide, and there is still no effective treatment for it [1,4,5]. In Asia, radical treatments such as surgery and liver transplantation are available for early-stage liver cancer, but the 5-year recurrence rate of HCC after hepatectomy is as high as 70%, even in patients with a single tumor ≤ 2 cm [6]. The 5-year recurrence rate after liver transplantation is only 10%–15%, but the extreme scarcity of liver sources cannot make liver transplantation the primary treatment method [6]. In addition, most patients with HCC are usually diagnosed at an advanced stage and are no longer eligible for curative treatment, and most patients can only be treated with drugs [7]. First-line drugs for advanced liver cancer, such as Sorafenib, Apatinib, and Bevacizumab, can significantly improve patient prognosis. Unfortunately, patients easily develop resistance to these drugs, which has a significant adverse effect on treatment [8–10]. This phenomenon also occurs in a variety of cancers, such as breast cancer [11], non-small cell lung cancer [12], and pancreatic cancer (PC) [13], so addressing tumor resistance is critical to the treatment and prognosis of cancer patients.

After searching for the keywords “tumor” and “drug resistance” on the PubMed website and reading a large number of related literature on tumor drug resistance, this review found that the emergence of tumor drug resistance is closely related to lactic acid modification. Recent studies have shown that histone lactylation alters chromatin structure and transcription, driving cancer progression and leading to drug resistance [14]. In addition, lactylation regulates immune cell function and promotes immunosuppression and tumor immune escape, which further complicates treatment. Therefore, we have integrated recent articles on tumor drug resistance and lactylation modification in the hope of elucidating the specific mechanisms between the two.

## 2 Biological Basis of Lactylation Modification

### 2.1 Discovery and Significance

Since its discovery in 1780, lactic acid has often been mistakenly thought to be a metabolic waste product under low-oxygen conditions and has a variety of harmful effects [15]. Later, the lactate shuttle hypothesis proved that lactate could be used both as an energy source and as a signaling molecule [16]. In the 1920s, Otto Warburg made the groundbreaking observation that tumors consume more glucose than surrounding normal tissue. This led him to come up with the concept of aerobic glycolysis, the process by which glucose is fermented into lactic acid instead of carbon dioxide in an oxygen-rich environment. This mechanism is currently referred to as the Warburg effect [17].

Lactylation was first described in 2019, when researchers discovered that histone proteins can be modified by emulsion groups under conditions of high glycolytic activity [18]. This modification has attracted great attention in epigenetics and cellular metabolism studies due to its role in cellular metabolism and its involvement in the regulation of various biological processes, especially in cancer cells [19–21]. Influencing protein function through lactylation is essential for cells to adapt to metabolic changes, especially in rapidly proliferating tumor cells exhibiting the Warburg effect (where aerobic glycolysis predominates). This discovery establishes a direct link between metabolism and epigenetic regulation, highlighting lactylation as a novel mechanism of cellular response to metabolic changes [19]. Unlike other Protein Post-translational Modifications (PTMs), such as acetylation or methylation, which rely on intermediates such as acetyl-CoA or S-adenosylmethionine, lactylation is uniquely tied to lactate metabolism, further reinforcing its role in linking energy production to cellular function [20]. Lactylation is now recognized as a significant regulatory mechanism with significant implications for gene expression, immune response, cell differentiation, and various disease pathologies.

### 2.2 Biochemical Mechanisms

#### 2.2.1 Lactate Source and Donor Role

The basis of lactate modification is the production of lactic acid and its role as an acyl donor:

(1) Lactate production by glycolysis pathway: In the process of cell metabolism, especially under anaerobic or high sugar conditions, glucose is converted into pyruvate through the glycolytic pathway, and then reduced to lactate under the action of lactate dehydrogenase (LDH) [22]. (2) Intracellular accumulation of lactic acid: Lactic acid can enter cells through monocarboxylic acid transporters (MCTs) or be directly produced by intracellular metabolism, and the level of lactate is significantly increased in the tumor microenvironment, inflammation, and hypoxia, providing sufficient substrates for lactylation modification [19]. (3) Formation of lactyl-CoA: Lactate can form Lactyl-CoA under the mediation of Coenzyme A (CoA), which is similar to the formation of acetyl coenzyme A (Acetyl-CoA) and provides an acyl donor for the lactylation of lysine residues [23].

#### 2.2.2 Catalytic Mechanism of Lactylation Modification

**Figure 1:**
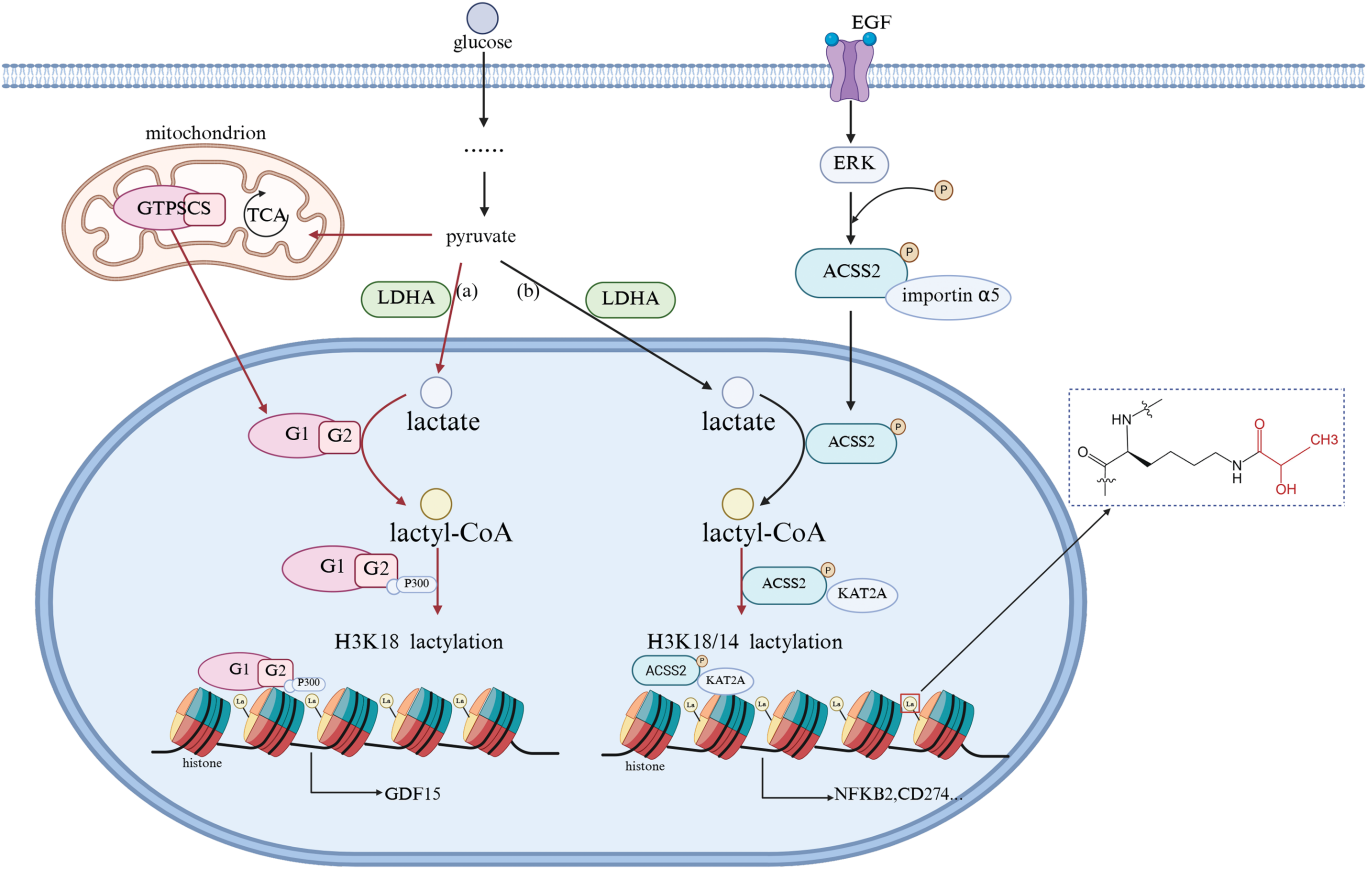
Molecular Link between Intracellular Lactate and Lactylation. EGF, epidermal growth factor; ERK, extracellular signal-regulated kinase; LDHA, lactate dehydrogenase A; ACSS2, Acetyl-CoA synthetase 2; GTPSCS, GTP-dependent succinyl-CoA synthetase in mammalian mitochondria. (The figure was made using Biorender)

Lactylation modification usually occurs on lysine (Lys) residues, and lactylation occurs when lactic acid groups covalently attach to the lysine residues of proteins, similar to acetylation, affecting protein function and interactions [19]. This process is often driven by an increase in intracellular lactate levels, which are often in response to hypoxia or enhanced glycolytic activity. Current research suggests that lactylation modification may be a spontaneous chemical reaction, but it may also be catalyzed by specific acyltransferases. For example, Fig. 1a: GTP-dependent succinyl-CoA synthetase in mammalian mitochondria (GTPSCS) can enter the cell nucleus through the nuclear localization signal of its G1 subunit, promote the synthesis of lactyl-CoA in the cell nucleus with lactate as a substrate, and enhance the interaction with the acyltransferase P300 (which transfers acetyl groups to histone lysine tails) through the acetylation modification at the K73 site of the G2 subunit, cooperatively regulating the level of histone H3 lysine 18 lactylation (H3K18la), upregulating the expression of the pro-cancer protein GDF15 [24]. Fig. 1b: After being activated by binding with epidermal growth factor (EGF), the epidermal growth factor receptor (EGFR) induces the extracellular signal-regulated kinase (ERK) to promote the phosphorylation of Acetyl-CoA synthetase 2 (ACSS2). Once phosphorylated, ACSS2 binds with importin α5 and undergoes nuclear translocation, entering the cell nucleus. Using lactate as a substrate, it enhances the production of lactyl-CoA within the nucleus. Additionally, it binds with lysine acetyltransferase 2A (KAT2A) to acetylate histone tyrosine, regulating the H3K18/14la levels and promoting the expression of NFKB2 and CD274 [25].

#### 2.2.3 Removal Mechanism of Lactylation Modifications

The reversibility of lactylation modifications is still being explored, and no specific delactylase has been identified. Studies have shown that some deacetylases (e.g., SIRT family) may have delactylation functions, such as SIRT3 and SIRT6, which play a role in the removal of histones and acylation modifications of metabolic enzymes, but the specific role of lactylation has not been fully understood [26–28]. The discovery of specific delactylating enzymes in the future will further reveal the modularity of lactylation modifications and their dynamic changes in cellular homeostasis.

### 2.3 Functional Role of Lactylation

#### 2.3.1 Gene Regulation and Epigenetic Control

One of the most important discoveries about lactylation is its role in gene expression. Histone lactylation has been shown to activate gene transcription by altering chromatin structure [18,29,30]. Unlike acetylation, which enhances gene activation primarily by neutralizing histone charge, lactylation is associated with persistent transcriptional activation of genes involved in immune response and metabolic adaptation. For example, it was demonstrated that lactylation could drive the switch of macrophages from a pro-inflammatory (M1) to an anti-inflammatory (M2) state [31,32]. This shift is essential for tissue repair and immune resolution, highlighting the role of lactylation in immune regulation. The ability of lactylation to modulate long-term gene expression patterns suggests that it may be involved in Immune memory (Immune memory—comprising T cells, B cells, and plasma cells and their secreted antibodies—is crucial for human survival. It enables the rapid and effective clearance of a pathogen after re-exposure, to minimize damage to the host) [33] and cellular adaptations to metabolic changes.

#### 2.3.2 Cell Differentiation and Development

In addition to its effects on gene transcription, lactylation is also involved in the process of cell differentiation. Studies have shown that stem cells use lactylation to regulate gene expression programs in specific lineages [34]. For example, in embryonic and hematopoietic stem cells, lactylation affects the differentiation pathway and determines cell fate decisions, which are essential for tissue development and homeostasis [34–36]. The role of lactylation in neural differentiation has also been explored, and studies have found that lactate-mediated alterations affect neurodevelopmental genetic programming [37]. Given the brain’s high metabolic demands and reliance on lactate produced by glycolysis, lactylation may be a key regulatory mechanism in neurogenesis and synaptic plasticity [38]. In addition, lactylation plays a crucial role in regulating the transcriptional programs required for tissue repair and homeostasis in muscle regeneration.

#### 2.3.3 Effects on Metabolic Pathways

**Figure 2:**
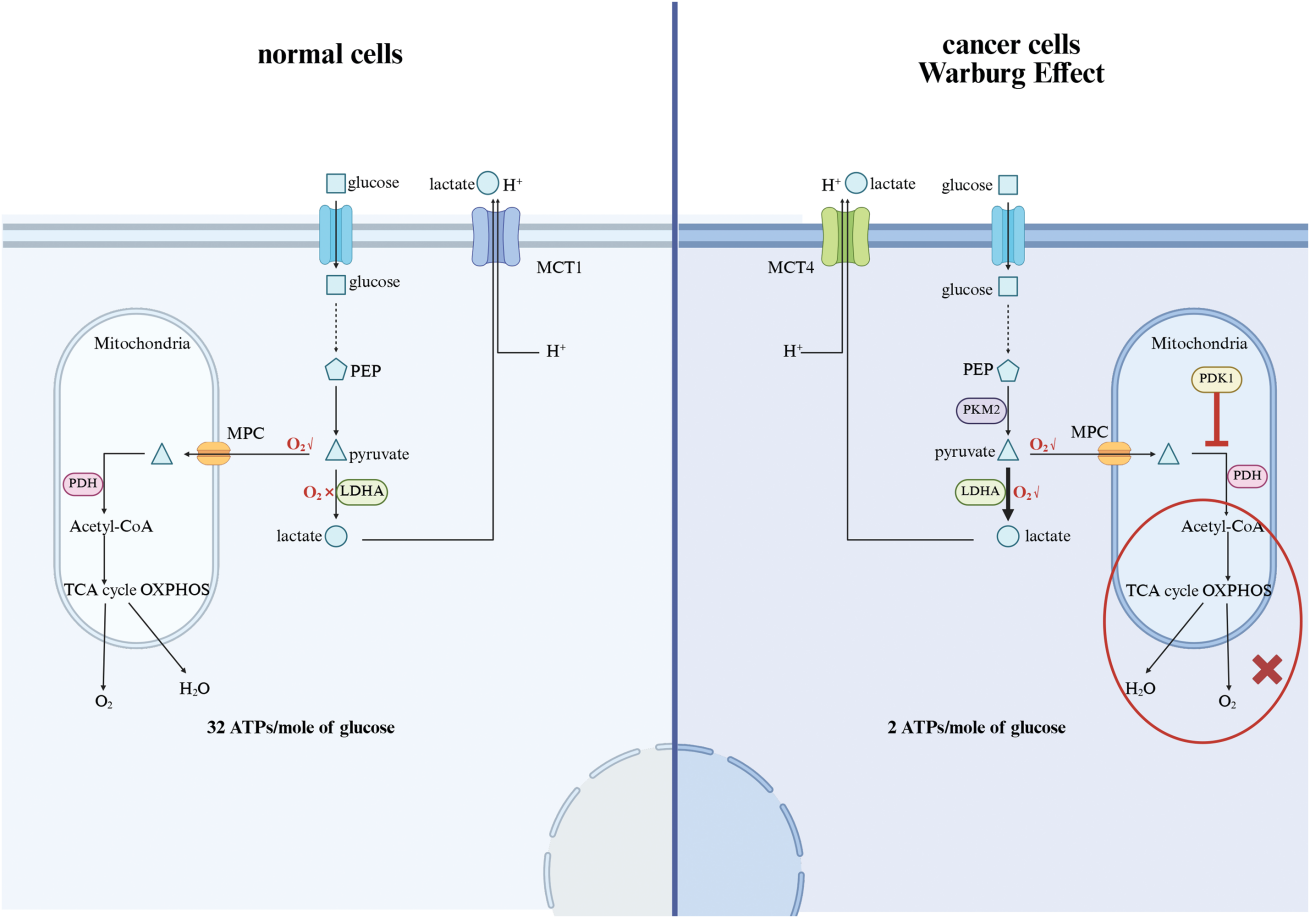
The mechanism of the Warburg effect and the impact on cancer. MCT1, Monocarboxylate Transporter 1; PEP, Phosphoenolpyruvate; MPC, Mitochondrial Pyruvate Carrier; PDH, Pyruvate Dehydrogenase; LDHA, Lactate Dehydrogenase A; PKM2, Pyruvate kinase M2; PDK1, Pyruvate dehydrogenase kinase 1; TCA, tricarboxylic acid cycle; OXPHOS: Oxidative phosphorylation (The figure was made using Biorender)

Because lactylation is intrinsically related to lactate metabolism, its role in metabolic regulation is of particular interest. Studies have shown that lactylation may affect metabolic enzyme activity, thereby regulating key pathways such as glycolysis, oxidative phosphorylation, and fatty acid metabolism [39]. By altering the function of metabolic enzymes, lactate fermentation may provide a mechanism for cells to fine-tune energy production in response to changes in environmental conditions. The Warburg effect in cancer metabolism manifests as cancer cells preferentially consuming glucose and relying on glycolysis rather than oxidative phosphorylation to generate ATP, even under aerobic conditions. The metabolic changes resulting from the Warburg effect are as follows [17,40]: Due to the expression of proto-oncogenes and the suppression of tumor suppressor genes in cancer cells, the transcription of low-activity pyruvate kinase M2 (PKM2), mitochondrial pyruvate dehydrogenase kinase 1 (PDK1), monocarboxylate transporter 4 (MCT4), etc., is activated. The expression of PKM2 limits the conversion of phosphoenolpyruvate to pyruvate, leading to an increase in the backup of glucose carbons, i.e., intermediates, above pyruvate kinase, which are shunted into various pathways for material synthesis, allowing continuous proliferation and development of tumor cells; PDK1 expression inactivates pyruvate dehydrogenase (PDH), and the mitochondrial tricarboxylic acid cycle and oxidative phosphorylation are inhibited, causing cancer cells to primarily undergo glycolysis even under aerobic conditions, i.e., aerobic glycolysis, and the excessive expression of lactate dehydrogenase A (LDHA) in cancer cells leads to the accumulation of lactate, which is transported outside the cell via MCT4 to participate in the formation of the tumor microenvironment, aiding in immune evasion; finally, since the rate of cytoplasmic ATP generation is approximately 100 times that of mitochondria, even though aerobic glycolysis yields only 2 moles of ATP per glucose molecule, as long as there is sufficient extracellular glucose supply, the ATP supply per unit time is higher than that of oxidative glucose metabolism, providing ample energy supply for cancer cells, and the significant increase in the demand for ATP in cancer cells also accelerates aerobic glycolysis (Fig. 2). At present, strategies to reduce lactate levels, inhibit lactate production, or regulate lactate-dependent gene expression are being explored as possible interventions for malignancies characterized by metabolic dysregulation [41].

### 2.4 Relationship between Lactylation and Disease

Lactylation is biologically significant in a variety of disease settings, particularly in cancer and immune diseases [42]. In cancer cells, increased glycolytic flux leads to increased lactate production, which in turn enhances histone lactylation and regulates gene expression patterns favorable to tumor progression. This metabolic-epigenetic interaction has been observed in various malignancies, including breast, colorectal, and glioma [43]. Excess lactate can enter the nucleus and induce histone lactylation, and this modification is catalyzed by the p300 enzyme, altering gene expression to promote tumor growth, for example, elevated histone H3 lysine 18 lactylation (H3K18la) levels in bladder cancer cells promote the expression of key transcription factors YY1 and YBX1, in which YBX1 plays a role in promoting DNA repair; YY1 can upregulate DNA repair genes (such as PTEN and Rad51), multidrug resistance genes (MDR1/ABCB1), and others, together leading to the chemotherapy resistance of tumor cells [44]. In addition, lactate also affects immune cells, especially macrophages, inducing their transformation into M2-type tumor-associated macrophage (TAM) phenotypes, thereby promoting immunosuppression and helping tumors escape immune surveillance [45].

## 3 Mechanisms of Tumor Drug Resistance

Cancer drug resistance is a major obstacle to effective cancer treatment, and its resistance mechanism can be broadly divided into intrinsic resistance and acquired resistance [46]. Numerous studies have demonstrated that both inherent and acquired tumor resistance significantly undermine the effectiveness of treatment [47]. To date, a variety of tumor resistance mechanisms have been studied and confirmed.

### 3.1 Tumor Cell Drug Target Mutations

The development of drug resistance, driven by mutations in drug targets, is one of the most daunting challenges in cancer treatment. Many targeted therapies work by binding to specific proteins whose activity drives tumor progression, and mutations within these target proteins can alter drug binding affinity, rendering the drug ineffective. One of the most well-documented examples is the emergence of secondary mutations in the BCR-ABL fusion protein in chronic myeloid leukemia (CML), which confers resistance to tyrosine kinase inhibitors (TKIs) such as imatinib. Mutations such as T315I, Y253H, and E255K within the kinase domain reduce the binding capacity of imatinib, perpetuating BCR-ABL signaling [48]. Similarly, resistance to epidermal growth factor receptor (EGFR) inhibitors in non-small cell lung cancer (NSCLC) is often caused by an EGFR T790M mutation that restores ATP affinity and reduces drug binding. These mutations are not random, but are often positively selected under drug stress, highlighting the dynamic evolutionary processes within the tumor during treatment [49]. In addition to kinases, resistance mutations in hormone receptors, such as ESR1 in estrogen receptor-positive breast cancer, have also been widely documented. Mutations in ESR1, especially in the ligand-binding domain, can lead to constitutive receptor activation even in the absence of estrogen, thereby undermining the efficacy of endocrine therapy [11]. These target mutations not only disrupt drug-target interactions but also alter downstream signaling, often reconnecting the signaling network to maintain oncogenic programs, illustrating the profound impact of drug-target mutations on treatment failure.

### 3.2 Inhibition of the Apoptotic Pathway

Inhibition of the apoptotic pathway involves complex interactions of molecular mechanisms, including regulation of Bcl-2 family proteins, inhibition of caspase activity, and activation of survival pathways such as PI3K/AKT signaling [50]. Among these mechanisms, overexpression of anti-apoptotic proteins such as Bcl-2 and Bcl-xL plays a key role in promoting cell survival by preventing extramitochondrial mitochondrial outer membrane permeabilization (MOMP) and cytochrome c release. Activation of the PI3K/Akt signaling pathway, which promotes cell survival and growth by phosphorylating downstream targets such as Bad and caspase-9, is common in malignancies, thereby preventing the initiation of apoptosis. In addition, inhibition of initiator and executioner caspases, such as caspase-8 and caspase-3, is a hallmark of apoptotic resistance in cancer cells. This resistance not only promotes tumor progression but also confers resistance to chemotherapeutic agents that target the apoptotic pathway.

### 3.3 Autophagy

Autophagy is a catabolic process responsible for the degradation of cellular components and plays a dual role in cell survival and cell death. Inhibition of autophagy is achieved by inhibition of key autophagy-related genes (ATGs) and the mammalian target of rapamycin (mTOR) signaling pathway, a central regulator of cell growth and autophagy [51]. The mTOR complex, especially Mechanistic Target of Rapamycin Complex 1 (mTORC1), is a key regulator of autophagy inhibition. Activation of mTORC1 inhibits the UNC-51-like kinase 1 (ULK1) complex, which is essential for autophagosome formation. This inhibition is commonly observed in cancer cells, where overactive mTOR signaling inhibits autophagy and enhances cell proliferation. In addition, Beclin-1 protein is a key regulator of autophagy initiation and is negatively regulated by Bcl-2, linking autophagy inhibition to apoptosis resistance [52].

### 3.4 Tumor Microenvironment (TME)

The TME is a complex and dynamic ecosystem that plays a vital role in tumor initiation, progression, metastasis, and response to treatment. It is made up not only of cancer cells but also of various stromal cells, including fibroblasts, endothelial cells, pericytes, immune cells, and an abundance of extracellular matrix (ECM), all of which are embedded in the evolving biochemical environment [53]. The cellular composition and biochemical properties of TME vary depending on tumor type and stage, but certain hallmarks, such as hypoxia, low pH, and immunosuppression, are common to many malignancies. For example, cancer-associated fibroblasts (CAFs) can modulate tumor behavior by secreting cytokines, remodeling the ECM, and directly interacting with tumor cells to enhance invasion and resistance to treatment [54]. TME also promotes angiogenesis, allowing tumors to establish their blood supply, which further supports their metabolic needs and dissemination potential. Importantly, interactions between immune cells, including tumor-associated macrophages (TAMs), regulatory T cells (Tregs), and bone marrow-derived suppressor cells (MDSCs), generate an immunosuppressive niche that protects tumors from immune surveillance and promotes immune evasion [55]. This intricate network of cellular crosstalk, biochemical gradients, and mechanical forces forms a permissive and protective environment that sustains cancer progression and severely limits treatment efficacy.

### 3.5 Epigenetics

A growing body of evidence suggests that epigenetic modifications can alter gene expression without altering the underlying DNA sequence. These modifications are particularly important in the setting of resistance to chemotherapy and targeted therapy. One of the most studied mechanisms is methylation of CpG islands, which can lead to tumor suppressor gene silencing or oncogene activation, leading to cancer development and treatment failure [56,57]. Aberrant DNA methylation can affect multiple pathways involved in cell cycle regulation, apoptosis, and DNA repair, allowing cancer cells to survive therapeutic stress. In addition to DNA methylation, histone modifications, such as acetylation and methylation, also play a crucial role in regulating chromatin structure and accessibility. Epigenetic alterations not only contribute to the acquisition of drug resistance but also mediate the survival of cancer stem cells (CSCs) [58]. CSCs are subsets of cells within tumors that can self-renew and differentiate into different cell types, and they are generally more resistant to conventional therapies than bulk tumor cells; therefore, CSCs are considered to be the root cause of tumor recurrence and metastasis.

## 4 The Relationship between Lactylation and Tumor Drug Resistance

A large number of studies have demonstrated that lactylation is one of the core mechanisms driving cancer drug resistance. It promotes metabolic reprogramming, enhances DNA repair, regulates the immune microenvironment, and modulates autophagy by lactifying specific targets of histones and non-histones (Table 1). Due to the Warburg effect, tumor cells preferentially produce energy through anaerobic glycolysis, even in the presence of oxygen, resulting in the accumulation of lactic acid in tumor cells as a by-product of glycolysis. The accumulation of lactate in the tumor microenvironment plays a key role in driving the lactylation process, which in turn drives these resistance mechanisms.

**Table 1:** Lactifying specific targets

Category	Target name	Description of the function	References
Histone species	H3K9la	Induction of glioblastoma (GBM) resistance to temozolomide (TMZ) through the promotion of LUC7L2 transcription, inhibition of MLH1 splicing, and reduction of mismatch repair (MMR) system activity.	[59]
	H3K18la	Activation of transcription factors YBX1 and YY1 enhances cisplatin resistance in bladder cancer cells.	[44]
	H4K12la	Inhibiting Schlafen Family Member 5 (SLFN5) expression promotes the malignant progression and drug resistance of Triple-negative breast cancer (TNBC).	[60]
Non-histone species	NSUN2	The NOP2/Sun domain family, member 2 (NSUN2) lactylation promotes its enzymatic activity to catalyze 5-methylcytosine modification on target GCLC mRNA, leading to upregulated Glutamate-Cysteine Ligase Catalytic Subunit (GCLC) expression and Glutathione (GSH) synthesis, rendering cancer cells resistant to ferroptosis.	[61]
	IGF2BP3	Upon lactification, Insulin-like Growth Factor 2 mRNA-Binding Protein 3 (IGF2BP3) stabilizes both PCK2 and NRF2 mRNA, augments the activity of the antioxidant system, and bolsters the resistance of hepatocellular carcinoma (HCC) to lenvatinib.	[62]
	ENSA-K63la	Activation of the Signal Transducer and Activator of Transcription 3 (STAT3)/C-C motif chemokine ligand 2 (CCL2) pathway generates an immunosuppressive microenvironment, thereby resisting therapy from immune checkpoint inhibitors.	[63]
	METTL3	The process of lactylation significantly increases the expression of Methyltransferase-like 3 (METTL3), thereby enhancing the resistance of acute promyelocytic leukemia to all-trans retinoic acid (ATRA).	[64]
	p53	p53 (K120/K139) is mediated by Alanyl-TRNA Synthetase 1 (AARS1) for lactylation, inhibiting DNA binding and transcriptional activity, thereby weakening its anti-tumor function.	[65]
	NBS1 (K388)	Lactylation modification enhances the interaction between NBS1 and MRE11, promoting the formation of the MRN complex, strengthening DNA homologous recombination repair, and leading to chemotherapy resistance.	[66]
	AK2 (K28)	Lactylation modification inhibits Adenylate Kinase 2 (AK2) function and promotes the proliferation and metastasis of hepatoma cells, which may be related to drug resistance.	[67]
	CCNE2 (K348la)	Lactylation of CCNE2 promotes proliferation, migration, and invasion of hepatocellular carcinoma cells and enhances resistance to chemotherapeutic agents.	[28]

### 4.1 Lactylation Modification Promotes Metabolic Reprogramming

**Figure 3:**
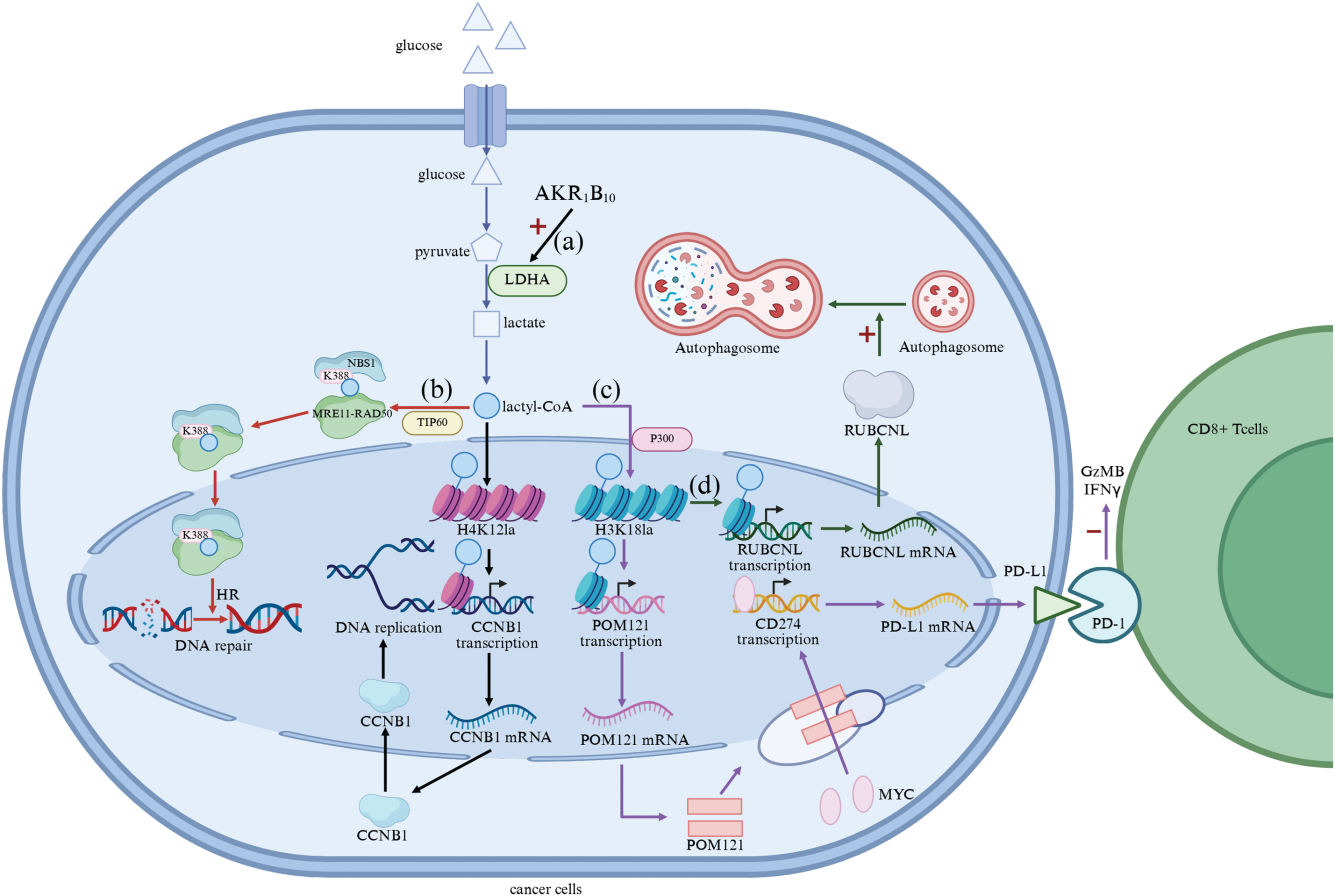
(**a**) Lactate Facilitates Metabolic Rewiring; (**b**) Lactylation promotes DNA repair; (**c**) Modulation of immune microenvironment by lactylation; (**d**) Regulation of autophagy by lactylation modification. AKR1B10, Aldehyde Ketone Reductase Family 1 B10; LDHA, Lactate Dehydrogenase A; CCNB1, cyclin B1; POM121, Pore Membrane Protein 121; PD-L1, Programmed cell Death 1-ligand 1; PD-l, Programmed Death-1 (The figure was made using Biorender)

One of the main contributions of lactylation to drug resistance is through enhanced glycolysis, a key metabolic pathway that provides energy to tumor cells under stressful conditions. Metabolic reprogramming characterized by increased glycolysis is a hallmark of tumorigenesis and resistance in various cancers. For example, cancer stem cells (CSCs) are a small subpopulation within tumors that have the ability to self-renew and generate new tumor cells. These cells are generally more resistant to conventional therapies and contribute to tumor recurrence. Lactylation plays a key role in maintaining the metabolic state of CSCs, which are highly dependent on glycolysis and rely on lactate for energy production. By promoting the expression of glycolytic enzymes and cell cycle regulators through histone lactylation, lactate supports the survival of CSCs under stressful conditions, including those triggered by chemotherapy [68,69]. In addition, aldehyde ketone reductase family 1 B10 (AKR1B10) has been shown to promote the Warburg effect in brain metastases of lung cancer, resulting in acquired resistance to the chemotherapeutic agent pemetrexed (PEM), which crosses the blood-brain barrier [70]. AKR1B10 enhanced glycolysis by upregulating LDHA, which converted pyruvate into lactate, leading to increased lactate production. This accumulation of lactate served as a precursor for histone lactylation, particularly H4 lysine 12 lactylation (H4K12la), which facilitated the activation of cell-cycle genes such as CCNB1, ultimately enhancing DNA replication and cell-cycle progression (Fig. 3a). This upregulation of glycolysis and histone lactylation driven by AKR1B10 was a key determinant in resistance to PEM, further exemplifying the central role that lactylation plays in metabolic reprogramming and drug resistance.

### 4.2 Lactated Modification Promotes DNA Repair

Lactylation also affects DNA repair mechanisms, which are essential for the survival of cancer cells under therapeutic stress. In many cancers, the DNA damage response (DDR) pathway is upregulated to repair DNA lesions induced by chemotherapy and radiation. Lactate-induced lactylation has been shown to enhance the repair of DNA double-strand breaks by promoting the activity of DNA repair proteins. For example, in gastric cancer cells, the lactylation of NBS1 K388 is catalyzed by TIP60 on lactyl-CoA, promoting the formation of the NBS1-MRE11-RAD50 trimeric MRN complex and the accumulation of HR repair proteins at DNA double-strand break sites. Through the MRN complex sensing DNA double-strand breaks (DSB), activating DNA repair pathways, and the function of HR repair proteins, DNA repair in cancer cells is enhanced, producing drug resistance [66] (Fig. 3b). In glioblastoma, lactylation of the X-ray Repair Cross Complementing 1 (XRCC1) protein, a key player in DNA repair, enhances its ability to repair DNA damage, thereby contributing to the development of resistance to chemoradiotherapy. This enhanced DNA repair capacity allows cancer cells to develop resistance to the genotoxic effects of the chemotherapy drug temozolomide (TMZ) [71]. Enzymes that inhibit lactate production or involve lactylation may provide a novel strategy to sensitize tumors to DNA-damaging agents, especially in cancers where lactylation plays a key role in maintaining genomic stability.

### 4.3 Lactylation Modification Regulates the Immune Microenvironment

In addition to promoting metabolic reprogramming and enhancing DNA repair, lactylation also regulates the immune microenvironment, aiding in immune evasion and resistance to immunotherapy. Tumors often reprogram the immune microenvironment to evade immune surveillance, in which lactate plays a key role [20]. In the tumor microenvironment (TME), high levels of lactate suppress the function of immune cells, particularly cytotoxic T cells, and promote the polarization of immunosuppressive cells such as Tregs and M2 macrophages [31,32]. Lactylation further contributes to immune evasion by stabilizing immune checkpoint proteins and promoting the accumulation of Tregs in the TME. For example, in NSCLC, histone lactylation (3HK18la) activates the transcription of pore membrane protein 121 (POM121), which enhances the MYC nuclear translocator. The elevated levels of nuclear MYC bind directly to the CD274 promoter, inducing PD-L1 expression, significantly inhibiting Granzyme B (GzMB) and Interferon-γ (IFNγ) levels in cocultured Cytotoxic T lymphocytes (CTLs), attenuating the cytotoxicity of CTLs, and contributing to the immune escape of NSCLC cells [72,73] (Fig. 3c). This lactate-induced immunosuppression is a major obstacle to the effectiveness of immune checkpoint inhibitors, and targeting the lactylation pathway may help overcome resistance to immunotherapy by restoring immune function and promoting anti-tumor immunity.

### 4.4 Lactylation Modification Regulates Autophagy

Autophagy is a cellular mechanism that maintains homeostasis by degrading damaged organelles and proteins [74]. In cancer cells, autophagy is often upregulated in response to therapeutic stress, allowing the cells to survive harsh conditions. Lactate-induced lactylation has been shown to promote the expression of autophagy-related genes, which helps cancer cells adapt to nutrient deprivation and survive chemotherapy. For example, in metastatic colorectal cancers (CRC), histone H3 lysine 18 lactylation (H3K18la) promotes the transcription of RUBCNL genes, enhancing autophagy through autophagosome maturation and contributing to CRC progression [75] (Fig. 3d).

## 5 Clinical Significance of Lactylation and Tumor Drug Resistance

### 5.1 Reveal the Mechanism of Drug Resistance and Provide New Ideas for Drug Resistance Reversal

Lactylation contributes to drug resistance by modifying both histone and non-histone proteins, thereby regulating gene expression and activating key signaling pathways associated with chemoresistance, immunosuppression, and metabolic adaptation. Notably, studies in hepatocellular carcinoma, prostate cancer, melanoma, and lung cancer have demonstrated how lactylation drives specific resistance phenotypes, such as stabilizing Insulin-like Growth Factor 2 mRNA-Binding Protein 3 (IGF2BP3), promoting Wnt/β-catenin signaling, or expanding Tregs [62,73,76,77].

These examples collectively illustrate how lactylation-driven mechanisms operate across different tumor types, often converging on shared pathways such as metabolic rewiring, immune evasion, and enhanced survival signaling. By mapping these mechanisms, this review highlights lactylation as a core hub linking metabolic stress to therapeutic resistance. This understanding provides a theoretical foundation for developing rational combination therapies aimed at reversing resistance and improving treatment efficacy across multiple cancers.

### 5.2 It has Become an Important Biomarker for the Prediction and Stratification of Tumor Treatment Effects

The level of lactylation modification is closely related to the response of tumor cells to chemotherapy, targeted therapy, and immunotherapy. Some studies have found that elevated lactylation levels are closely related to poor prognosis in patients, especially in immune checkpoint inhibitor treatment populations, and the high expression of lactylation-modifying proteins in tumor tissues may indicate the formation of an immunosuppressive microenvironment, which in turn affects treatment response [78,79]. Therefore, lactylation-related molecules such as histone H3 lysine 18 lactylation (H3K18la) [76], IGF2BP3 [62], and NSUN2 [80] are expected to be used as predictors of efficacy or patient typing tools to assist in precision treatment.

### 5.3 Provide New Targets and Combination Therapy Strategies for Anti-Drug Resistance Therapy

The formation of lactylation modification depends on the high lactate state in tumor cells, and targeting lactate production and lactylation-related enzymes (such as LDHA, p300, etc.) becomes an important strategy to reverse drug resistance. Studies have shown that LDH inhibitors such as FX11 can reduce lactate levels, reduce lactylation modifications, and restore chemotherapy sensitivity [81]; At the same time, the combination of metabolic modulators and existing chemotherapy/targeted drugs has demonstrated synergistic anti-tumor effects in a variety of tumor models [82]. In response to the immunosuppressive effect driven by lactylation modification, it can also form synergies with programmed death receptor 1 (PD-1)/ programmed death ligand 1 (PD-L1) inhibitors to enhance anti-tumor immune response and overcome immunotherapy resistance [73,83,84]. In addition, the role of lactylation in regulating angiogenesis and metastasis is a key process that supports tumor growth and metastasis. Lactylation has been shown to regulate the expression of angiogenic factors such as fibroblast growth factor 2 (FGF2) through lactated transcription factors such as YY1 [85]. This lactylation enhanced the transcriptional activity of YY1, which led to increased FGF2 expression and might be one of the mechanisms underlying its promotion of tumor angiogenesis. In addition, lactylation affects the metastatic potential of tumor cells by regulating the expression of proteins involved in cell migration and invasion. Therefore, targeted therapies targeting the lactylation pathway that regulates angiogenesis and metastasis may provide a novel strategy to inhibit tumor growth and prevent cancer from spreading to other organs. This approach may be particularly beneficial in cancers known to have positive metastatic behavior, such as breast [86], prostate [87], and lung [88] cancers.

### 5.4 Promote the Development of Individualized Treatment Strategies

Lactylation modification has certain plasticity and dynamics, which are regulated by multiple factors such as metabolic state, acidity of microenvironment, and partial pressure of oxygen. Therefore, the development of a strategy of “dynamic monitoring + precise intervention” based on lactylation modification-related markers will help to achieve individualized anti-drug resistance therapy in the true sense. For example, through the dynamic monitoring of lactylation modification profile, the patient’s response to a treatment strategy can be assessed in real time, and the treatment plan can be adjusted in time to improve the efficacy and avoid unnecessary toxic side effects.

## 6 Limitations and Challenges of Current Research

Although lactylation has attracted considerable attention as a post-translational modification affecting cancer biology, there are still some challenges that hinder its comprehensive understanding and clinical application in cancer drug resistance. One of the main limitations is the lack of precise and standardized techniques to measure lactylation in biological samples. In contrast to more established PTMs such as acetylation or phosphorylation, lactylation is still in the exploratory stage, and the tools for detecting and quantifying this modification have not been fully developed. Current methods, such as western blotting and mass spectrometry, still need to be further optimized to reliably measure lactylation in a variety of tissue types, especially clinical samples. In addition, the lack of universal antibodies against lactylated proteins complicates the study of this modification in different cancers, limiting its application in clinical settings.

Another challenge is the complexity of explaining the biological significance of lactylation. Although lactylation has been shown to play a role in cancer progression and drug resistance, its exact mechanism remains unclear. The dual role of lactylation as both a metabolic signal and an epigenetic regulator makes it difficult to determine whether it is primarily involved in tumorigenesis, progression, or resistance. For example, studies have shown that lactylation of histones can regulate gene transcription and promote tumor survival, but it remains uncertain whether lactylation is a major driver of these processes or simply reflects secondary metabolic changes [21]. More comprehensive functional studies are needed to determine the exact role of lactylation in various cellular pathways and to assess its impact on chemoresistance.

The lack of established biomarkers of lactylation-related modifications is also an obstacle to its clinical application. While certain lactylation-related features, such as those found in triple-negative breast cancer (TNBC) [89,90] and pancreatic adenocarcinoma [91,92], show potential to predict patient outcomes, but they are still in their early stages. For example, the lactylation-related gene signatures identified for TNBC, while promising, have not been validated in larger clinical cohorts or applied to treatment decisions. In addition, lactylation-related features often overlap with other molecular features, such as glycolysis and hypoxic pathways, which makes it difficult to attribute changes in drug resistance specifically to lactylation without further unraveling these interrelated mechanisms.

## 7 Future Research Directions

As the understanding of the role of lactylation in cancer drug resistance continues to advance, the review has identified three promising future research directions.

First, develop reliable, standardized methods to quantify lactylation in a clinical setting. Advancing the sensitivity and specificity of these assays is critical to translating lactylation studies into therapeutic contexts. More accurate quantification of lactylation modifications, especially in tumor samples, will help better understand and target lactylation pathways in cancer.

Second, further research is needed on the molecular mechanisms by which lactylation affects cancer progression and drug resistance. While lactylation has been shown to modulate key proteins involved in DNA repair and immune regulation, its full role in different cancer types remains unclear. For example, how lactylation interacts with other post-translational modifications, such as acetylation and methylation, to drive tumorigenesis remains to be fully elucidated. Understanding these interactions is critical to determining the precise role of lactylation in the molecular network that regulates cancer cell survival and drug resistance. In addition, the potential of lactylation in modulating tumor cell plasticity and metastasis needs to be explored further, as these processes are central to treatment failure.

Finally, since lactylation is associated with metabolic reprogramming, targeting lactate metabolism provides a new therapeutic strategy to overcome drug resistance. Recent studies have shown that targeting lactate production and lactylation offers the potential to sensitize tumor cells to chemotherapy and immunotherapy [93,94]. Combining lactate metabolism inhibitors, such as LDHA inhibitors, with traditional cancer treatments may enhance treatment effectiveness and reduce the development of resistance [94]. In addition, understanding how lactylation affects immune cell function opens up new avenues for immunotherapy, as lactate-mediated lactylation in immune cells may promote immunosuppression in the tumor microenvironment. Targeting these metabolic and epigenetic pathways represents a promising strategy to improve cancer treatment outcomes and address the challenge of drug resistance.

## 8 Conclusion

In conclusion, lactylation provides a novel and promising target for cancer therapy, especially in the context of drug resistance. Further study of the molecular mechanisms of lactylation, its role in various cellular processes, and its potential as a therapeutic target will provide valuable insights into overcoming cancer treatment resistance. Combining strategies that target lactylation with existing treatments can significantly improve treatment outcomes and provide new avenues for personalized cancer care.

## Data Availability

Not applicable.

## Abbreviations

HCC: Hepatocellular carcinoma
PC: Pancreatic cancer
NSCLC: Non-small cell lung cancer
PTMs: Protein post-translational modifications
LDH: Lactate dehydrogenase
LDHA: Lactate dehydrogenase A
MCTs: Monocarboxylic acid transporters
CoA: Coenzyme A
PKM2: Pyruvate kinase M2
PDK1: Pyruvate dehydrogenase kinase 1
MCT4: Monocarboxylate transporter 4
PDH: Pyruvate dehydrogenase
H3K18la: Histone H3 lysine 18 lactylation
TAMs: Tumor-associated macrophages
CML: Chronic myeloid leukemia
TKIs: Tyrosine kinase inhibitors
EGFR: Epidermal growth factor receptor
MOMP: Mitochondrial outer membrane permeabilization
ATGs: Autophagy-related genes
TME: The tumor microenvironment
ECM: Extracellular matrix
CAFs: Cancer-associated fibroblasts
MDSCs: Marrow-derived suppressor cells
CSCs: Cancer stem cells
AKR1B10: Aldehyde ketone reductase family 1 B10
H4K12la: H4 lysine 12 lactylation
DDR: DNA damage response
DSB: DNA double-strand breaks
TMZ: Temozolomide
Tregs: T cells
POM121: Pore membrane protein 121
CRC: Colorectal cancer
APOC2: Apolipoprotein C-II
FGF2: Fibroblast growth factor 2
TNBC: Triple-negative breast cancer
